# Selenium-Enriched Microorganisms: Metabolism, Production, and Applications

**DOI:** 10.3390/microorganisms13081849

**Published:** 2025-08-07

**Authors:** Lin Luo, Xue Hou, Dandan Yi, Guangai Deng, Zhiyong Wang, Mu Peng

**Affiliations:** 1 Hubei Key Laboratory of Biological Resources Protection and Utilization, Hubei Minzu University, Enshi 445000, China; luolin0430@163.com (L.L.); hx17320056301@163.com (X.H.); 15565575786@163.com (D.Y.); m18785717523@163.com (G.D.); 2 College of Biological and Food Engineering, Hubei Minzu University, Enshi 445000, China

**Keywords:** selenium-enriched microorganisms, selenium metabolism mechanisms, selenium-enriched products, nano-selenium, biotransformation

## Abstract

Microorganisms, as abundant biological resources, offer significant potential in the development of selenium-enrichment technologies. Selenium-enriched microorganisms not only absorb, reduce, and accumulate selenium efficiently but also produce various selenium compounds without relying on synthetic chemical processes. In particular, nano-selenium produced by these microorganisms during cultivation has garnered attention due to its unique physicochemical properties and biological activity, making it a promising raw material for functional foods and pharmaceutical products. This paper reviews selenium-enriched microorganisms, focusing on their classification, selenium metabolism, and transformation mechanisms. It explores how selenium is absorbed, reduced, and transformed within microbial cells, analyzing the biochemical processes by which inorganic selenium is converted into organic and nano-selenium forms. Finally, the broad applications of selenium-enriched microbial products in food, medicine, and agriculture are explored, including their roles in selenium-rich foods, nano-selenium materials, and disease prevention and treatment.

## 1. Introduction

Selenium is an essential trace element required by the human body. Although needed in only small amounts, it plays a critical role in maintaining normal physiological functions. The biological activity of selenium is primarily attributed to its presence in selenoproteins [1], which are involved in various processes such as antioxidant defense [2], immune regulation [3], endocrine function [4], as well as cell proliferation and apoptosis [5]. Selenium supports the synthesis of key antioxidant enzymes such as glutathione peroxidase (GPx) and selenoprotein P [6], which eliminate free radicals and peroxides, thereby reducing oxidative stress and cellular damage [7]. In nature, selenium exists mainly in two forms: organic and inorganic. Organic selenium typically occurs in the form of selenium-containing amino acids, primarily selenomethionine (SeMet) [8] and selenocysteine (SeCys) [8]. Plants absorb inorganic selenium from the soil and convert it into these organic, which are then consumed by animals through plant-based foods or selenium-enriched feed [9]. In contrast, inorganic selenium is typically found as sodium selenate (Na_2_SeO_4_) or sodium selenite (Na_2_SeO_3_). However, excessive intake of inorganic selenium can be toxic, making its conversion into organic forms essential for improving both bioavailability and safety for human consumption [10].

Microorganisms are closely linked with selenium, as they are not only essential for microbial growth but also significantly influences microbial metabolic processes [11]. Among various transformation methods, microbial strategies are widely recognized as the most effective, environmentally friendly, and scalable approach for converting inorganic selenium into less toxic and more bioavailable forms [12]. A common method for producing selenium-enriched products involves supplementing microbial culture media with selenium salts, such as Na_2_SeO_3_ or Na_2_SeO_4_ to promote the biosynthesis of organic selenium compounds or selenium nanoparticles (SeNPs) [13]. Furthermore, microorganisms are also excellent candidates for use as food additives due to their ability to biotransform inorganic selenium into organic forms with higher nutritional value [14].

In recent years, selenium-enriched microorganisms have gained increasing attention in both scientific research and practical applications [12]. Through processes such as bioaccumulation and biotransformation, microorganisms can effectively absorb inorganic selenium from the environment and convert it into organic selenium compounds, including seleno-amino acids and selenoproteins, which are more easily absorbed and utilized by the human body [14]. Consequently, selenium-enriched microorganisms hold great promise not only in enhancing selenium bioavailability but also for their versatile applications in food, dietary supplements, agriculture, and medicine. Therefore, this review provides a comprehensive overview of recent advances in the classification of selenium-enriched microorganisms, their selenium metabolism and transformation mechanisms, production strategies, and multi-disciplinary applications. In addition, current challenges and future research directions in this emerging field are also discussed.

## 2. Bibliometric Analysis of Research on Selenium-Enriched Microorganisms (2002–2025)

**Figure 1:**
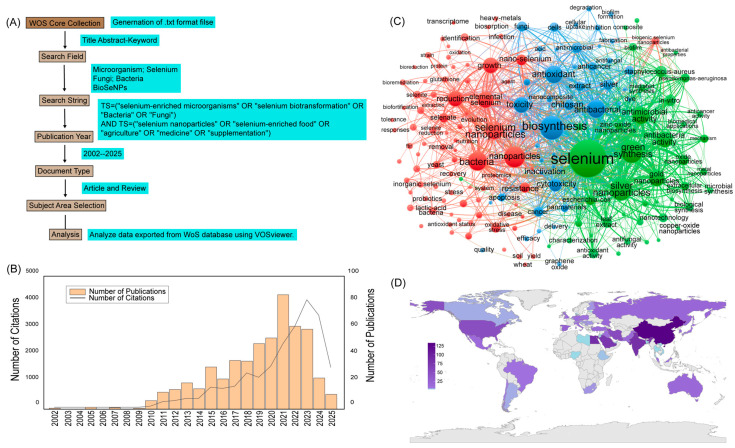
Overview of the bibliometric analysis process and research trends in the field of selenium-enriched microorganisms. (**A**) Flowchart of the literature retrieval strategy used for bibliometric analysis based on the Web of Science Core Collection. (**B**) Annual number of publications (2002–2025) related to selenium-enriched microorganisms. (**C**) Keyword co-occurrence network generated using VOSviewer. (**D**) Number of Publications on Selenium-Enriched Microorganisms by Country (2002–2025).

To assess the development and research focus within the field of selenium-enriched microorganisms, a bibliometric analysis was conducted using data retrieved from the Web of Science (WoS) Core Collection. Publications were identified using a combination of keywords, including “selenium-enriched microorganism”, “selenium biotransformation”, “selenium-enriched food”, and related terms (Figure 1A). The search was limited to articles and reviews published between 2002 and 2025, and the data were further analyzed using VOSviewer version 1.6.20.

The annual publication output, shown in Figure 1B, reveals a clear upward trend over the past two decades, with a marked increase in the number of publications after 2010. This trend indicates a growing scientific interest in the topic, particularly in recent years. However, the data also show a slight decrease in both the number of publications and citations after 2021–2023. Despite this, the peak in publication numbers during the last five years suggests that selenium-enriched microorganisms have emerged as a prominent research focus, driven by their potential applications in nutrition, functional food development, and biomedicine.

A keyword co-occurrence analysis was performed to identify research hotspots, as visualized in Figure 1C. The network map is composed of three major clusters. The green cluster, centered on keywords such as selenium, biosynthesis, and nanoparticles, represents research efforts related to the microbial transformation of selenium and the synthesis of SeNPs. The red cluster includes terms such as yeast, strain, and fermentation, highlighting studies that focus on microbial species selection and optimization of culture conditions. The blue cluster, containing terms like antioxidant activity, toxicity, and health benefits, reflects investigations into the bioactivity, safety, and therapeutic potential of selenium-enriched microbial products. The global distribution of research on selenium-enriched microorganisms is presented in Figure 1D. The map highlights research output by country, with darker shades indicating a higher volume of publications. Significant contributions are evident from countries in North America, Europe, and Asia, indicating a growing international interest and active research collaboration in this field.

Collectively, this bibliometric analysis underscores the expanding interdisciplinary interest in selenium-enriched microorganisms. The research landscape is increasingly integrating fields such as microbiology, nanotechnology, food science, and medicine, emphasizing the importance and versatility of microbial selenium transformation in both fundamental research and applied biotechnology.

## 3. Types of Selenium-Enriched Microorganisms

Selenium-enriched microorganisms refer to microbial species capable of growing in selenium-containing media and accumulating selenium within their cells. Through specific metabolic pathways, these microorganisms can convert inorganic selenium into organic selenium compounds or SeNPs, which exhibit enhanced bioavailability and nutritional value for both humans and animals [15]. Selenium-enriched microorganisms mainly include bacteria, yeasts, and fungi.

### 3.1. Selenium-Enriched Bacteria

Selenium-enriched bacteria can metabolize inorganic selenium species—such as selenate or selenite—into biologically active organic forms, therefore reducing selenium toxicity and improving its safety and efficacy in nutritional applications [12]. Representative selenium-enriched bacterial genera include *Lactobacillus* [16], *Bacillus* [17], *Actinomycetes* [18], and *Pseudomonas* [19]. Among these, *Lactobacillus* and *Bacillus* have been most extensively studied due to their high selenium-conversion efficiency, application potential, and metabolic stability [17,20]. *Lactobacillus* is widely recognized for its roles in gut health and immune modulation [20], while *Bacillus* species are valued for their environmental resilience and versatility in industrial applications [21].

#### 3.1.1. Selenium-Enriched Lactic Acid Bacteria

Lactic acid bacteria (LAB) are widely used in the production of probiotic products. Various strains from genera such as *Lactobacillus*, *Lactococcus*, *Streptococcus*, *Pediococcus*, and *Bifidobacterium* have demonstrated the ability to transform inorganic selenium into organic selenium compounds and SeNPs [16]. The efficiency of selenium conversion and resistance to selenium toxicity varies significantly among different genera and species. Different microbial strains exhibit varying selenium conversion efficiencies and tolerances to selenium toxicity, as shown in Table 1. These LAB can metabolize selenite and selenate into compounds such as SeCys, SeMet, and methylselenocysteine (MeSeCys) [22], or into elemental selenium (Se^0^) [23]. A study by Zhang et al. [24] reported that *Bifidobacterium animalis* 01 could absorb 16.7–39.6% of the inorganic selenium in the culture medium and convert the most of it into organic forms. The distribution of organic selenium within the cell was as follows: 50.7–63.0% in proteins, 9.62–18.7% in polysaccharides, 0.273–0.754% in nucleic acids, and 20.8–30.9% in other components. LC-MS analysis confirmed that SeMet was the major selenium compound in the protein fraction. Additionally, selenium-enriched LAB have been shown to produce chaperone proteins during fermentation, enhancing their stress resistance [25]. Selenium-enriched LAB exhibit both high selenium-conversion capacity and antimicrobial activity. They can disrupt the cellular structures of pathogenic microbes and inhibit their growth [12]. In vivo studies indicate that selenium bioaccumulated by *Bifidobacterium* can convert approximately 50% of inorganic selenium into organic selenium, thereby supporting selenium homeostasis in animal models [24]. Selenium plays a key role in redox reactions and the metabolism of selenocompounds, contributing to the host’s physiological stability.

Furthermore, selenium-enriched *Lactobacillus* strains have demonstrated various biological activities. For example, selenium-enriched *Lactobacillus plantarum* has shown antioxidant and anti-inflammatory effects. It mitigates the harmful impact of alkaline stress in carp by modulating gut microbiota composition, enhancing intestinal barrier integrity, reducing systemic toxin translocation, and improving blood metabolite profiles [26]. In selenium-deficient regions, the inclusion of selenium-enriched LAB in animal feed has been shown to enhance selenium content in animal products, thus improving the nutritional value of meat, milk, and eggs [27]. Research by Che et al. [28] demonstrated that dietary supplementation with selenium-enriched *Lactobacillus plantarum* alleviated salt stress-induced growth inhibition and hepatic damage in carp by modulating cytokine expression and antioxidant gene activity. Moreover, selenium-treated LAB exhibited antibacterial activity against *Escherichia coli* and *Staphylococcus aureus* [29], highlighting their potential use as functional food additives.

**Table 1:** Selenium Reduction Capacity and Tolerance Concentrations of Different Lactic Acid Bacteria Strains.

Strain Name	Selenium Conversion Capacity	Maximum Tolerable Concentration	Ref.
*Streptococcus thermophilus* CCDM 144	SeCys, SeMet	10 mg/L	[30]
*Enterococcus faecium* CCDM 922 A	SeCys, SeMe	10 mg/L	[30]
*L. plantarum* HBUT121	selenoprotein	2 mg/L	[31]
*Pediococcus acidilactici* ATCC 8042	SeCys, SeNPs	1 mg/L	[32]
*L. paracasei* ML13	SeCys	150 mg/L	[33]
*L. paracasei* CH135	SeCys	150 mg/L	[33]
*Enterococcus faecium* ABMC-05	SeCys	100 mg/L	[34]
*Lactobacillus delbrueckii* ssp. *bulgaricus*	SeNPs	80 mg/L	[35]
*Streptococcus thermophilus*	SeNPs	80 mg/L	[35]
*E. faecium* CCDM 922A	SeCys	100 mg/L	[36]
*Lcc. lactic* subsp. *cremoris* CCDM 72	SeCys	100 mg/L	[36]
*Lcc. lactic* subsp. *cremoris* CCDM 73	SeCys	100 mg/L	[36]
*L. plantarum* CXG-4	Se^0^	16 mg/L	[14]
*Limosilactobacillus fermentum* CGMCC 17434	organic selenium	12 mg/L	[37]
*Streptococcus thermophilus* CICC6220	organic selenium	16 mg/L	[38]
*Bifidobacterium breve* CICC6184	organic selenium	10 mg/L	[38]
*Levilactobacillus brevis* CRL 2051	SeCys, SeNPs	0.15 mg/L	[39]
*Fructobacillus tropaeoli* CRL 2034	SeCys, SeNPs	0.15 mg/L	[39]
*L. plantarum* L123	Se^0^	15 mg/L	[40]
*L. delbrueckii* subsp. *bulgaricus* CCDM 364	SeCys, SeNPs	50 mg/L	[41]

#### 3.1.2. Selenium-Enriched *Bacillus* spp.

*Bacillus* species are well-known for their ability to utilize inorganic selenium as a source for bioaccumulation. Chen et al. [21] optimized the fermentation conditions for *B. cereus*, reporting a remarkable selenium conversion rate of 94.3 ± 0.2% under optimal conditions, including 7% inoculum size, 33 °C incubation temperature, and 170 rpm shaking speed. These conditions facilitated efficient production of organic selenium, especially selenium-binding proteins, which are particularly suitable for functional applications. In addition to selenium bioaccumulation, selenium-enriched *Bacillus* strains have also demonstrated significant heavy metal-binding capabilities. Shang et al. [42] showed that *B. subtilis* enriched with selenium markedly reduced lead (Pb) accumulation in fish tissues, alleviated oxidative stress, and mitigated lead-induced toxicity in carp. Further research by Lu et al. [43] explored the molecular mechanisms underlying the neuroprotective effects of selenium-enriched *B. subtilis* in goldfish exposed to perfluorohexanoic acid (PFHxA). Their findings indicated that selenium-enriched *Bacillus* suppressed reactive oxygen species (ROS) production and apoptosis by activating the BDNF/PI3K/AKT/GSK-3β signaling pathway. This highlights the therapeutic potential of selenium-enriched *Bacillus* species in neurotoxicology and environmental health contexts.

Similarly, Xue et al. [44] demonstrated the protective role of selenium-enriched *Bacillus* against environmental contaminants such as PFHxA in aquaculture, providing insights into the intricate interplay between diet, gut microbiota, and host metabolism in aquatic organisms exposed to environmental pollutants. Bioactive metabolites derived from selenium-enriched *Bacillus* species, including selenium-bound peptides, have shown promising pharmacological properties, such as antioxidant, anti-inflammatory, and anti-tumor activities [45]. Among these, SeNPs have attracted significant attention due to their high biocompatibility, low toxicity, and potential for targeted drug delivery [46]. Consequently, SeNPs are being actively explored as promising candidates for the development of anticancer therapeutics, antimicrobial agents, and immune modulators [47].

#### 3.1.3. Other Selenium-Enriched Bacteria

Currently, various other bacterial species are also capable of accumulating and reducing selenium. One such anaerobic bacterium, *Thauera selenatis*, was the first isolated strain proven to possess selenate respiratory reductase activity [48]. *Sulfurospirillum barnesii* is a microaerophilic, Gram-negative, thermophilic bacterium that can reduce selenate to selenite and Se^0^ [48]. In addition, a facultative anaerobic bacterium, *Azospira* sp. A9D-23B, has been identified as an efficient selenate-reducing strain, capable of collecting extracellular SeNPs from selenium-containing wastewater [49]. *Enterobacter cloacae* can also reduce selenite under both aerobic and anaerobic conditions and produce red Se^0^ extracellularly [48].

Many bacteria in soil also possess the ability to accumulate and reduce selenium. *Metasolibacillus* sp. ES129 and *Oceanobacillus* sp. ES111, isolated from selenium-rich soils, exhibit high reduction rates and tolerance to selenite. When the selenite concentrations were 4.24 mM and 4.88 mM, respectively, ES129 and ES111 achieved the highest efficiency in converting selenite to SeNPs [50]. Another strain isolated from selenium-rich soil, *Rhodococcus qingshengii* PM1, also demonstrates high selenium tolerance—with selenite resistance up to 100 mM—and is capable of reducing 99% of 50 mM selenite within 72 h [51]. Anna V. Tugarova et al. [52] observed through experiments that *Azospirillum thiophilum* can synthesize SeNPs and conducted spectral analyses on them. Additionally, *Rhizobium* species in soil can promote nitrogen accumulation in leguminous plants [53].

In addition, the actinomycete *Streptomyces griseobrunneus* strain FSHH12 can synthesize SeNPs and catalytically degrade bromothymol blue dye via photocatalysis [18]. *Kitasatospora* sp. SeTe27, also exhibits high tolerance to selenite [54].

Among *Pseudomonas* species, Zhang et al. [19] found that combined treatment with DL-alanine and *Pseudomonas aeruginosa* can promote the transformation of Se^0^ by the bacterium into SeNPs, thereby reducing cadmium toxicity in soil. Moreover, *Pseudomonas frigusceleri* MPC6 can reduce selenite through the formation of SeNPs, contributing to the bioremediation of both marine and freshwater environments [55].

### 3.2. Selenium-Enriched Fungi

#### 3.2.1. Selenium-Enriched Molds

Selenium-enriched fungi, particularly *Aspergillus* and *Trichoderma*, are among the most representative types of selenium-enriched microorganisms. Gao et al. [56] found that in *Aspergillus oryzae* A02, selenium exists primarily as selenium proteins and SeNPs, with selenium content being 2–3 times higher than that found in selenium-enriched yeast. Upon exogenous selenium enrichment, *Monascus* (Red Yeast) demonstrated the following distribution of organic selenium in its cells: selenium proteins (28.04–47.33%), selenium polysaccharides (3.80–5.07%), and selenium nucleic acids (1.84–3.07%) [57]. These fungi are capable of absorbing inorganic selenium from the culture medium through extracellular enzymes and metabolic pathways, subsequently converting it into organic selenium compounds such as selenium amino acids (e.g., SeMet and SeCys) and SeNPs. Additionally, selenium-enriched *A. oryzae* A02 has been shown to stabilize the gut microbiota in male mice, regulate lipid metabolism, modulate α-linolenic acid levels, and promote testosterone biosynthesis. Thus, *A. oryzae* A02 is considered a promising nutritional regulator with potential to enhance reproductive function [58].

#### 3.2.2. Selenium-Enriched Mushrooms

Several edible fungi, including shiitake mushrooms (*Lentinula edodes*) [59] and golden oyster mushrooms (*Pleurotus citrinopileatus*), are capable of accumulating selenium [60]. Compared to non-enriched mushrooms, selenium-enriched mushrooms exhibit enhanced anticancer, antioxidant, antibacterial, and anti-inflammatory properties, largely due to the abundance of selenium polysaccharide complexes [61]. For instance, the mycelial selenium polysaccharides in *Oudemansiella radicata* have shown excellent antioxidant activity both in vitro and in vivo [62]. Among different selenium sources, oyster mushrooms demonstrate the highest selenium uptake efficiency, with organic selenium content ranging from 46% to 90% in these mushrooms, making them an excellent dietary source of selenium [63]. The application of 2,4-D as a growth enhancer for oyster mushrooms can increase their organic selenium content by as much as 8.03 times [64]. Analysis of white (*Pleurotus ostreatus*) and pink (*Pleurotus djamor*) oyster mushrooms revealed that the white variety primarily contains SeMet, while the pink variety contains SeMet, SeCys, and MeSeCys [65]. Moreover, selenium enrichment does not negatively affect the mushrooms’ ability to absorb other metal elements such as zinc (Zn), copper (Cu), calcium (Ca), and magnesium (Mg) [66].

Additionally, selenium-enriched *Stropharia rugoso-annulata* mycelial extract [67], *Agaricus bisporus* [68], and selenium-enriched shiitake mushrooms (*L. edodes*) [69] have all demonstrated strong antioxidant properties. Selenium-enriched shiitake mushrooms, rich in nutrients, also exhibit significant anticancer effects, making them a valuable source of dietary selenium. Furthermore, purified selenium-enriched *Cordyceps polysaccharides* (SeCPS) [70] and a novel selenium-enriched *C. polysaccharide* (SeCPS-II) [71] have shown anticancer activity, suggesting their potential as therapeutic agents. Moreover, Chen et al. [72] proposed that the high-molecular-weight proteins in selenium-enriched mushrooms may not be SeMet-containing proteins but rather proteins bound to SeMet.

#### 3.2.3. Selenium-Enriched Yeast

Selenium-enriched yeast is one of the most commonly used selenium-rich fungi, produced by cultivating yeast in selenium-enriched media to accumulate selenium [73]. A key feature of selenium-enriched yeast is its ability to convert inorganic selenium into organic selenium compounds, particularly SeMet and SeCys [8]. These organic selenium compounds exhibit higher bioavailability, making selenium-enriched yeast widely used in the development of dietary supplements, functional foods, and health products. Research has demonstrated that the organic selenium content in *Saccharomyces cerevisiae* ranges from 1 to 4.5 mg/g dry weight, with 58% of selenium present as MeSeCys, with a final concentration reaching 5.746 mg/g [73,74]. In another selenium-enriched yeast, *Candida utilis*, the organic selenium content is 1.0 mg/g [75]. Studies on fermentation conditions for selenium-enriched yeast have revealed that fermenting with 100 μg Se at 8 °C for 150 h provides the best selenium retention in wine [76]. Due to its ability to accumulate high levels of selenium, yeast cells often store excess inorganic selenium [8], which is assimilated through sulfur metabolism [77]. This process leads to the formation of selenium-enriched cells containing various organic selenium compounds.

Compared to plants, yeast contains higher protein levels [78]. Yeast can accumulate selenium in various forms via both intracellular and extracellular biological accumulation [79]. Inorganic selenium is biotransformed into organic forms, which not only reduces toxicity but also enhances its absorption [79,80]. Selenium influences yeast metabolism [81], and the addition of selenium-enriched yeast [82] and selenium peptides from yeast autolysates [83] can improve dough texture, making it softer with higher creep compared to traditional dough. Selenium-enriched yeast is highly biocompatible, with excellent selenium bioavailability [84]. Its enrichment process is relatively simple and controllable, preventing toxicity issues arising from excessive selenium accumulation. Furthermore, selenium-enriched yeast effectively eliminates *Aspergillus* species, preventing fruit contamination [85]. By adjusting fermentation conditions, selenium content in yeast can be precisely controlled to meet food safety standards. Selenium-enriched yeast is also highly nutritious, containing selenium, proteins, B vitamins, and other trace elements [84].

## 4. Selenium Metabolic Mechanisms in Selenium-Enriched Microorganisms

### 4.1. Absorption and Reduction of Inorganic Selenium

Microorganisms absorb inorganic selenium from their environment primarily in the form of selenite and selenate [86]. Although these inorganic selenium compounds serve as a primary selenium source, they can be toxic. Thus, microorganisms must reduce these compounds into less toxic or non-toxic forms to mitigate their harmful effects [12]. Wang et al. [15] elucidated the reduction mechanisms of selenate and selenite, as well as the biosynthesis mechanisms of biogenic selenium nanoparticles (BioSeNPs) during these processes.

**Figure 2:**
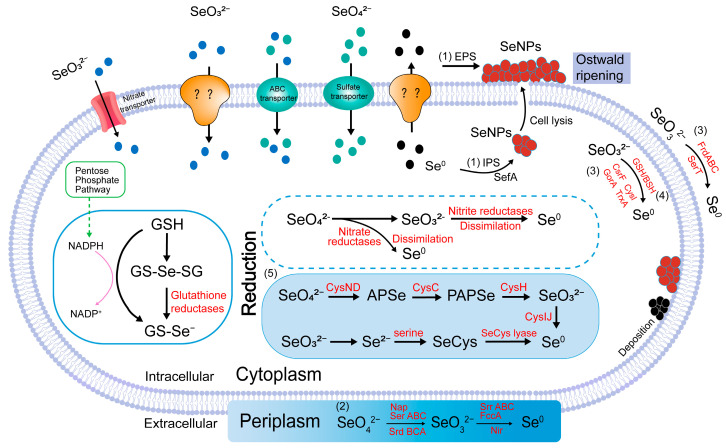
Metabolic Pathway of Selenium in Bacteria. (1) EPS: Extracellular Polymeric Substances; IPS: Intracellular Polymeric Substances; SefA: Secreted Protein. (2) Se(VI) can be anaerobically reduced in the periplasm to Se(IV) by nitrate reductase and respiratory selenate reductase, respectively. Se(IV) can then be further anaerobically reduced to Se^0^ in the periplasm by respiratory Se(IV) reductase (SrrABC), fumarate reductase (FccA), and nitrite reductase (Nir). (3) Se(IV) can be aerobically reduced to Se^0^ by multiple reductases in the cytoplasm, or (4) by thiols such as GSH and BSH. (5) CysNDCHIJ: Sulfate Reductase. APSe, PAPSe: The reduction of selenate to SeCys is a key intermediate in the formation of biologically active selenium compounds.

The first stage in the metabolism of selenium involves the transport of selenate anions into the microbial cell (Figure 2). Selenate is transported via sulfate permeases or oxyanion transport proteins [87,88]. Once inside the cell, the reduction of selenate and selenite occurs. The complete mechanism of this reduction, however, remains incompletely understood. There are two main reduction mechanisms [89]. The first occurs in anaerobic microorganisms, where selenate and selenite undergo dissimilatory (respiratory) reduction to Se^0^. The second mechanism involves the oxidation of organic substrates or hydrogen (H_2_) and the dissimilatory reduction of selenium oxyanions. Selenate and selenite can be reduced by nitrate reductase and nitrite reductase through dissimilation to Se^0^ [90], with the resulting SeNPs either deposited near the cell membrane or released from the cell through cell lysis [16]. In bacteria, the reduction of selenate and selenite involves multiple enzymatic systems. Selenate is reduced to selenite by respiratory selenate reductase (Ser) and nitrate reductase in the periplasmic space. Ser is the product of an operon that controls its expression, which includes genes encoding three structural polypeptides (SerABC) and a fourth gene (serD). The SerABC operon encodes structural proteins involved in the formation of SeNPs, while serD encodes a chaperone protein that assists in the proper assembly of SerA [48]. The Srd system is an essential enzyme complex for respiratory selenate reduction, with its function encoded by the SrdBCA operon. It facilitates electron transfer and directs the localization of signal peptides to the periplasm [48]. It is further converted into BioSeNPs through the action of SrrABC, fumarate reductase (FccA), Nir, and selenite reductase (SerT) [91]. The Srr system is a specialized enzyme complex for dissimilatory selenite reduction. Its core function is to reduce selenite (SeO_3_^2−^) to Se^0^, serving as a key metabolic mechanism that couples selenite reduction with bacterial growth [48]. Additionally, this process involves the hydrolysis of glutathione (GSH) and benzenesulfonyl groups (BSH), ultimately leading to the formation of BioSeNPs.

Some microorganisms utilize specialized selenium transport proteins or carrier proteins to transport selenite or selenate from the external environment into the cell [92]. These transport systems efficiently recognize and absorb selenium ions, ensuring an adequate selenium supply for the microorganism [93]. Additionally, microorganisms can absorb inorganic selenium through natural diffusion across the cell membrane. Given the structural similarity between selenate and sulfate [94], selenate or selenite can enter the cell through osmosis [95], a process that is energy-independent and efficient for absorbing small molecular ions.

**Figure 3:**
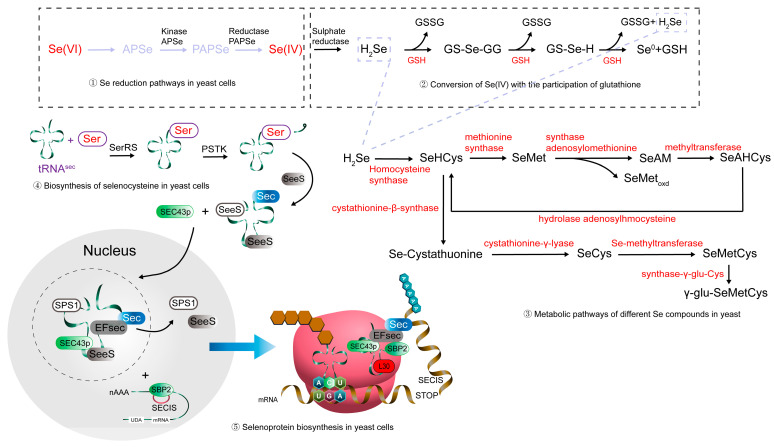
Metabolic pathway of selenium in yeast. (1) Selenium reduction pathways within yeast cells; (2) Conversion of Se(IV) with the involvement of glutathione; (3) Metabolic pathways of various selenium compounds in yeast; (4) Biosynthesis of selenocysteine in yeast cells; and (5) Selenoprotein biosynthesis in yeast cells. GSH: Glutathione; GSSG: Oxidized Glutathione; GS-Se-GG: A form of selenoglutathione (GS-Se); GS-Se-GG: A selenium-containing derivative of glutathione; Ser: Respiratory selenate reductase; SPS1: A key enzyme responsible for the synthesis of selenophosphate; EFsec: A factor involved in the translation elongation process; Sec: Selenocysteine; SBP2: A protein related to the insertion of selenocysteine, which assists in selenoprotein synthesis by binding to the SECIS element (Selenocysteine Insertion Sequence); L30: Ribosomal protein.

In yeast cells, the metabolism of selenium involves several key steps, beginning with the reduction of Se(VI) to Se(IV), which is catalyzed by kinases, APS reductase, and other reductases (Figure 3). Glutathione (GSH) plays a crucial role in this reduction process, as it participates in the conversion of Se(VI) to its active form, Se(IV), which can then be incorporated into selenoproteins. Following this, selenium is integrated into amino acids, particularly SeCys and SeMet, through complex biosynthesis pathways. The enzyme cystathionine-β-synthase is involved in the formation of SeCys, while methionine synthase and SeMet synthase facilitate the biosynthesis of SeMet. Additionally, SeCys is metabolized into various selenium compounds, including Se-methylselenocysteine (SeMeCys), by specific methyltransferase enzymes. SeCys is incorporated into selenoproteins in yeast cells, with the help of specialized selenocysteine tRNA (Sec-tRNA) and the Sec insertion machinery. This complex network of pathways ensures the efficient bioaccumulation and utilization of selenium, contributing to the production of bioactive selenium compounds essential for yeast growth and function.

### 4.2. The Generation of BioSeNPs

Certain microorganisms [12] reduce selenium oxyanions to elemental red selenium through intracellular or extracellular mechanisms, forming SeNPs [96]. These nanoparticles possess significant antioxidant activity and biocompatibility [97], making them highly valuable in various applications [98]. Traditional methods for synthesizing SeNPs often rely on chemical reductants, which can produce toxic by-products and result in challenges in controlling particle size and morphology [99]. In contrast, microorganisms such as bacteria and fungi can synthesize BioSeNPs through a green synthesis process, offering superior biocompatibility, lower toxicity, and broader application potential compared to traditional chemical methods [100].

BioSeNPs have shown promise in biomedicine, including in cancer treatment, targeted chemotherapy, molecular diagnostics, and drug delivery [46]. Compared to other forms of selenium, BioSeNPs exhibit lower toxicity, higher absorption efficiency, and enhanced antioxidant properties [101,102]. BioSeNPs are also effective adsorbents, capable of adsorbing heavy metal ions from soil and water, and facilitating the recovery of metal ions [103]. For example, Mao et al. [104] studied the beneficial effects and detoxification potential of BioSeNPs synthesized from guava extract in alleviating antimony (Sb) toxicity in rice seedlings. Their results demonstrated that BioSeNPs can reduce Sb accumulation in crops and improve crop yield under Sb stress.

Furthermore, BioSeNPs interact with proteins and other biomolecules containing functional groups (such as NH, C=O, COO, and C-N) found in microbial cells and plant extracts, conferring biological activity to the nanoparticles [105]. Research has shown that SeNPs synthesized using *Oryza sativa* spirulina SOSA-4 as a reducing and stabilizing agent exhibit significant antioxidant activity in assays such as DPPH, FRAP, SOR, and ABTS, along with excellent antibacterial properties [106], further supporting their potential as biocompatible nanomaterials. Additionally, SeNPs synthesized in yeast have demonstrated anti-ulcer activity, accelerating ulcer healing through antioxidant effects and modulation of gut microbiota imbalances associated with gastric ulcers [97].

Microorganisms can reduce inorganic selenium to SeNPs through intracellular metabolic reactions regulated by selenium reductase enzymes [14]. Selenium ions are reduced to elemental selenium and aggregate into nanoparticle forms. These nanoparticles may be encapsulated by specific proteins or lipid membranes within the microbial cell, further stabilizing them. Many bacteria and fungi also synthesize SeNPs extracellularly [97]. After reducing inorganic selenium to elemental selenium, the nanoparticles either accumulate on the cell surface or are secreted into the surrounding environment [50]. The formation of SeNPs is influenced not only by the microorganism’s reductase enzymes but also by the environmental conditions surrounding the cell, such as pH, temperature, and redox potential. SeNPs exhibit potent antioxidant activity, capable of scavenging free radicals, reducing oxidative damage, and providing protective effects to cells [107].

## 5. Production Process of Selenium-Enriched Microbial Products

### 5.1. Screening of Selenium-Enriched Microorganisms

The type of selenium salt used significantly affects the efficiency of selenium accumulation in microorganisms. Sodium selenite and sodium selenate are the most commonly used inorganic selenium sources; however, their reduction efficiencies within microorganisms can vary [108]. There are significant differences in the selenium tolerance levels among different microorganisms. The maximum tolerable concentration for *Pediococcus acidilactici* ATCC 8042 [32] is 1 mg/L, while *L. paracasei* CH135 [33] can tolerate up to 150 mg/L. Even recent studies have shown that *Halomonas* sp. can tolerate up to 1200 mM (=94.8 g/L) of selenite [109], which is the highest tolerance level reported to date. Therefore, selecting the appropriate selenium salt and concentration is crucial, depending on the microbial species and its capacity to convert selenium. The maximum selenium tolerance levels of some LAB are shown in Table 1. Additionally, *Lactobacillus delbrueckii* spp. [35] shows inhibited growth when the selenium concentration exceeds 80 mg/L. The selenium concentration must be optimized within a specific range, as both excessively high and low concentrations can negatively impact microbial growth and selenium conversion efficiency. For example, a study examining the effect of low selenium concentrations on selenium accumulation in *Lactobacillus plantarum* found that optimal selenium enrichment occurred when the sodium selenite concentration reached 4 mg/L, resulting in selenium accumulation of 1.89 mg/g [32]. Higher selenium concentrations (e.g., 20 mg/L) inhibited bacterial growth, while a concentration of 10 mg/L allowed *Lactobacillus* to maintain its integrity and exhibit enhanced selenium accumulation capacity [110]. However, when sodium selenite concentration exceeded a threshold, bacterial growth declined [111]. Additionally, treatment methods can affect the selenium accumulation abilities of microorganisms. For instance, ultrasonic treatment of *Saccharomyces cerevisiae* significantly increased selenium accumulation, with a 2.78-fold increase compared to untreated controls [112].

### 5.2. Optimization of Fermentation Conditions

The growth and selenium conversion activity of microorganisms are also affected by environmental pH and temperature [113]. Different microorganisms have their own optimal pH [112] and temperature ranges [114], and both excessively high or low pH and temperature can inhibit microbial metabolic activity [95]. Therefore, during production, it is crucial to precisely control the pH and temperature of the fermentation broth based on the characteristics of the target microorganism. Generally, temperatures between 20 °C and 40 °C and a pH range of 6.0 to 7.5 are considered optimal [39,115]. The growth and selenium conversion efficiency of selenium-enriched microorganisms are influenced by various fermentation conditions. To achieve high-yield selenium-enriched microbial products, these conditions need to be systematically optimized [38]. However, research on the optimization of fermentation processes remains somewhat limited. For instance, adding selenium-enriched germinated rye seeds to sourdough can promote the growth of LAB, reducing fermentation time by 8 to 16 h. This addition increases the selenium content of the final bread fivefold without negatively impacting its sensory quality [116]. Equally important is the selection of appropriate strains for selenium-enriched fermentation. In a study preparing selenium-enriched probiotics and enriching selenium in fermented fruit juices, *Lactobacillus plantarum*, *Streptococcus thermophilus*, and *Bifidobacterium animalis* were tested. The results indicated that *Streptococcus thermophilus* exhibited the best selenium tolerance, with a selenium enrichment rate of 33.8% [38].

## 6. Applications of Selenium-Enriched Microbial Products

### 6.1. Agricultural Field

**Figure 4:**
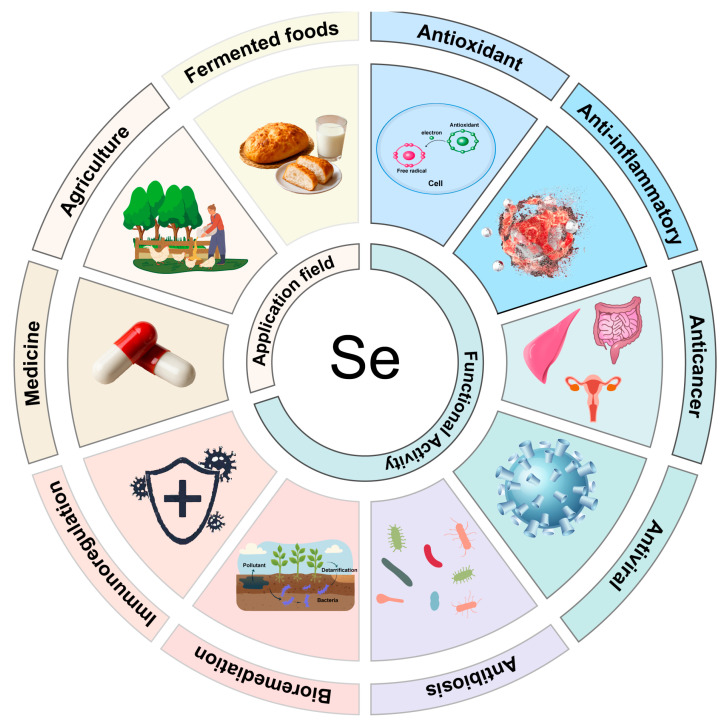
Functional Activity and Application Fields of Selenium-Enriched Microorganisms.

Selenium-enriched microbial products offer significant advantages in the agricultural sector (Figure 4). We have listed the selenium summaries of different types of selenium-enriched microorganisms (Table 2). They not only enhance the selenium content in crops but also improve plant disease resistance and overall plant health and growth [117]. By converting selenium into more bioavailable forms, these microorganisms promote selenium uptake by plant roots [118]. Selenium-enriched microorganisms help transform inorganic forms of selenium in the soil into organic selenium compounds that plants can utilize, thus increasing the nutritional value of crops [119]. For example, sodium selenite inhibits broccoli growth but using selenium-enriched yeast as an exogenous selenium fertilizer promotes growth and increases the selenium content. Under the treatment of selenium-enriched yeast, broccoli accumulates MeSeCys and SeMet within its tissues [120]. Although broccoli treated with selenium-enriched yeast is not directly edible, it can be processed into nutritional supplements. Similarly, using selenium-enriched yeast as a selenium source enhances cabbage growth. At a selenium concentration of 8 mg/kg, important compounds within the cabbage are maintained at high levels [121].

In addition to enhancing plant growth, selenium-enriched microorganisms can serve as bio-pesticides. Their metabolic products, such as selenoproteins or selenium polysaccharides, exhibit antimicrobial [122], antiviral [123], and antifungal [124] properties, offering effective pest and disease control. This reduces the need for chemical pesticides, protecting the ecological environment and enhancing the safety of agricultural products [125]. Selenium-enriched microorganisms are also valuable as bio-fertilizers and bio-feeds. Selenium-rich *Lactobacillus plantarum* has been shown to alleviate liver damage caused by chronic alkaline stress and modify the diversity of gut microbiota [26]. This bacterium regulates gut microbial balance by reducing the production and translocation of lipopolysaccharides, mitigating the negative effects of alkalinity stress on the liver. Selenium-enriched *Lactobacillus plantarum* could be explored as a feed additive in saline–alkali aquaculture.

**Table 2:** Selenium Summary of Selenium-Enriching Microorganisms.

Representative Strain	Product Type	Selenium Tolerance Concentration	Conversion Efficiency	Application Direction	Ref.
*Lactiplantibacillus plantarum* NML21	SeNPs, selenoproteins	4 mg/L	86.17%	Selenium-enriched yogurt, functional fermented dairy products	[20]
*Bifidobacterium animalis* 01	Selenoproteins	10 mg/L	77.4–86.6%	Intestinal regulation, selenium supplementation health products	[24]
*Bacillus cereus*	Organic selenium	150 mg/L	94.3%	Nutritional supplements, medicinal drugs	[21]
*Saccharomyces cerevisiae* EMY6#	SeMet, SeCys, SeMecys	30 mg/L	85.85–94.51%	Selenium-enriched beer, functional cider, feed	[126]
*Pleurotus ostreatus* CICC 50115	Organic selenium	30.65 mg/L	--	Functional edible mushrooms, medicinal polysaccharides	[64]
*Metasolibacillus* sp. ES129	SeNPs	733.56 mg/L	91%	Environmental remediation	[50]
*Rhodococcus qingshengii* PM1	SeNPs	17,294 mg/L	99%	Environmental remediation	[51]

Feeding selenium-enriched *Bacillus subtilis* to Nile tilapia has been found to improve fish growth, blood parameters, liver enzyme activity, and immune response [127]. Similarly, selenium-enriched yeast has been shown to enhance growth performance, liver and kidney histology, and economic benefits in juvenile fish when fed at a recommended dose of 3.98 mg/kg of diet [128]. Selenium-enriched yeast also improves antioxidant activity and fish meat quality when used as feed for Pomacanthus imperator [129] and yellow catfish [130]. In studies on juvenile sea cucumbers, supplementation with selenium-enriched yeast before and after mating improved growth, gut health, and immune status, while increasing selenium content in the body wall [131,132]. Feeding selenium-enriched yeast to egg ducks [133], layer hens [134], and broiler chickens [9] has been shown to enhance mineral deposition in the muscles of broiler chickens, improve antioxidant balance within the body, reduce plasma malondialdehyde levels, and increase selenium deposition in eggs and duck eggs, thereby enabling the production of selenium-enriched eggs.

Selenium-enriched microorganisms can also enhance plants’ resistance to environmental stresses such as drought, high temperatures, and heavy metal contamination, improving stress tolerance and disease resistance. *Bacillus* species, through selenium incorporation, regulate the expression of key metabolic pathway genes to initiate defense and detoxification responses to antimony (Sb(III)) [135], reducing the harmful effects of this heavy metal on crops. Additionally, selenium-enriched microorganisms promote the synthesis of antioxidant enzymes in plants, enhancing their antioxidant capacity and helping mitigate oxidative stress. Selenium-oxidizing bacteria can also lower cadmium (Cd) content in plants [136], further reducing the environmental impact of cadmium on soil and ecosystems.

### 6.2. Food Industry

In the food industry, selenium-enriched microbial products primarily focus on the production of functional foods. Selenium-enriched foods not only address the growing demand for nutrition and health but also increase the added value of food products. Common applications include the following:

In dairy production, selenium-enriched microorganisms, such as selenium-enriched *Lactobacillus* species, can be used in the fermentation of products like yogurt and cheese. Wang et al. [20] demonstrated that selenium-enriched *Lactobacillus plantarum* NML21 significantly regulates key metabolic pathways and promotes the accumulation of beneficial metabolites, making it suitable for the production of selenium-enriched functional yogurt. This selenium-enriched yogurt exhibited excellent physicochemical properties and antioxidant activity, meeting consumer demand for nutritional and health benefits. Deng et al. [137] isolated a strain of *Lactobacillus* (CGMCC No. 6683) from kefir grains that can survive at high selenium concentrations. This strain was pretreated with sodium selenite to produce selenium-enriched *Lactobacillus*. Additionally, a selenium-enriched strain, *Rhamnus* L20, was selected through experimental screening for its high tolerance to inorganic selenium and its efficient conversion rate. The conversion rate of inorganic selenium reached as high as 87.65%, and selenium-enriched probiotic goat milk tablets and goat milk powder developed from this strain showed long-term stability [138].

Selenium-enriched microorganisms are also suitable for producing fermented beverages. Selenium-enriched yeast and LAB can be used to produce selenium-rich functional drinks. For instance, *Lactobacillus casei* CRL 2051 was found to remain resistant to selenium-induced changes during fruit juice and milk fermentation [139]. This strain can be used to produce selenium-enriched fruit juices. A 250 mL serving of this beverage provides 64% of the recommended dietary intake for selenium, with 28% of it in the form of SeCys, a non-toxic and bioavailable compound that enhances nutritional value. Research by Wu et al. [140] showed that *Bacillus subtilis* is suitable for selenium enrichment. Selenium-enriched *Bacillus subtilis* ferments well in low-salt media, and its antimicrobial properties enhance the synergistic effects of selenium with antimicrobial peptides. This strain has been applied in the industrial production of fermented products such as soybean paste, kimchi, and soy sauce. Selenium-enriched yeast, when fermented with malt, can be used to produce selenium-enriched alcoholic and non-alcoholic beverages like selenium-rich beer and kvass [141]. Mutant yeast strains (EMY6#), can accumulate selenium up to 2616.92 mg/kg. When used to ferment apple cider, EMY6# not only increases the selenium content of the cider but also enhances the levels of polyphenols and aromatic compounds compared to regular fermentation [126]. In the fermentation of food, selenium-enriched *Lactobacillus* can reduce nitrite levels in kimchi by producing organic acids, thus mitigating the potential harmful effects of nitrites on human health [31]. Additionally, fermented chickpeas enriched with selenium using *Bacillus subtilis* [142] can serve as a functional food to help manage hypertension. Additionally, edible fungi are increasingly popular as functional foods due to their nutritional value and health benefits. Selenium-enriched edible mushrooms, such as shiitake mushrooms, can stimulate the biosynthesis of phenolic compounds and flavonoids, positively affecting free radical generation [143].

### 6.3. Pharmaceutical Field

Selenium-enriched microbial products are showing great promise in the pharmaceutical field, especially for their unique applications in antioxidant, anti-tumor, and immune-regulatory activities [16]. Selenium-enriched microbial components, such as selenium-containing proteins and polysaccharides, regulate the immune system by enhancing immune cell activity and immune responses. Yang et al. [144] found that *Lactobacillus rhamnosus GG* modulated lipid metabolism and oxidative stress-related genes in the liver of alcohol-excessive mice. This strain also upregulated the expression of proteins related to intestinal barrier function, improving alcohol-induced intestinal barrier damage. Selenium-enriched *Lactobacillus plantarum* ZZU8-12, isolated from healthy human feces, has been shown to regulate gut microbiota and protect against acute liver injury (ALI) by regulating short-chain fatty acid-producing bacteria in the gut microbiome [145]. Selenium-enriched lactobacilli also exhibit superior antibacterial activity against common foodborne pathogens, including *Salmonella Typhimurium*, *Escherichia coli*, *Staphylococcus aureus*, and *Listeria monocytogenes* [146], thus slowing their growth and disrupting cellular structures. Additionally, Hu et al. [147] found that selenium-enriched *Bifidobacterium longum* DD98, in combination with cyclosporine A (CsA), improved ulcerative colitis in mice while reducing renal toxicity. Yang et al. [148] co-cultured *Escherichia coli* with several probiotics (including *Candida*, *Lactobacillus acidophilus*, *Lactobacillus rhamnosus*, and *Streptococcus thermophilus*) and found that selenium-enriched probiotics significantly improved gut microbiota composition and exhibited the most potent antimicrobial effects. These probiotics effectively antagonized pathogens and enhanced the body’s antioxidant capacity. Selenium-enriched yeast has been found to mitigate chronic inflammation, such as in atherosclerosis, by downregulating pro-inflammatory genes and reducing inflammation [149]. It also helps reduce cadmium-induced nephrotoxic apoptosis in chickens through modulation of the miR-26a-5p/PTEN/PI3K/AKT signaling pathway [150].

Xue et al. [151] demonstrated through experimental studies that selenium-enriched *Bacillus subtilis* enhanced cognitive function in *Candida albicans*-infected subjects by activating the BDNF/TrKB/AKT/GSK-3β signaling pathway, thereby reducing brain damage caused by the infection. These findings offer valuable insights into the effects of selenium-enriched *Bacillus subtilis* on aquatic animals, suggesting its potential as a safe and beneficial selenium supplement. It not only increases the nutritional value of aquatic products but also contributes to reducing environmental pollution. Darwish et al. [152] investigated the side effects of piroxicam, a common drug used to treat pain, swelling, and stiffness in osteoarthritis and rheumatoid arthritis. Piroxicam was found to cause side effects such as hypertension, elevated liver enzymes, and hepatitis. However, the addition of a selenium-enriched *Bifidobacterium longum* BSe50/20/1 mutant strain was shown to repair liver and kidney damage, improve their function, reduce the expression of inflammatory genes, and increase the expression of anti-inflammatory genes, making it a promising agent for mitigating the side effects of piroxicam. Emamectin benzoate (EMB), a widely used insecticide, can lead to liver damage. Zhang et al. [47] reported that selenium-enriched *Bacillus subtilis* alleviated inflammation and significantly inhibited ferroptosis induced by EMB-induced liver damage, including the production of NF-κB, TNF-α, and IL-1β. Furthermore, *Bacillus subtilis* activated the Nrf2 signaling pathway, enhancing the cell’s antioxidant capacity, which effectively mitigated oxidative damage and inflammation, offering additional liver protection. This suggests that selenium-enriched *Bacillus subtilis* holds promise as a potential treatment for drug-induced liver damage. Atrazine (ATR), an herbicide, can induce oxidative stress and inflammation by inhibiting the Nrf2 pathway and activating the NF-κB pathway, leading to cell apoptosis. However, selenium-enriched yeast has been shown to reduce the toxicity caused by ATR by inhibiting oxidative stress [153,154]. In cancer treatment, selenium-enriched yeast has demonstrated the potential to increase oxidative stress, inhibit tumor cell growth, and induce apoptosis in human breast cancer cells, while sparing non-cancerous cells [155]. Additionally, the hydrolyzed selenium peptides from selenium-enriched yeast exhibit excellent antioxidant properties and may be utilized as additives in pharmaceutical and cosmetic products to mitigate oxidative skin damage [156].

With ongoing research into the biological mechanisms of selenium, the metabolic products of selenium-enriched microorganisms hold significant potential in drug development, particularly for treating chronic diseases, immune system disorders, and cancer.

## 7. Research Focus, Challenges, and Future Directions

Despite the promising capabilities of several microorganisms in enriching selenium and converting it into organic selenium compounds, the specific mechanisms underlying their selenium metabolism remain incompletely understood. Additionally, the exact mechanisms by which microorganisms absorb inorganic selenium across the cell membrane and subsequently reduce it into organic selenium compounds for metabolic use are a critical knowledge gap that needs to be addressed.

While selenium is an essential trace element for human health, excessive selenium intake can lead to selenium toxicity. Different forms of selenium compounds exhibit varying toxicity levels in the human body. For instance, SeNPs synthesized via traditional methods may pose toxic risks due to their unique physicochemical properties and by-products [104,157]. Evaluating the safety of different selenium forms and establishing safe dosages for practical applications remains a key research challenge.

Moreover, the long-term safety of selenium-enriched microbial products, particularly with prolonged consumption, is yet to be fully determined. Continuous intake of selenium-enriched foods or medicinal products may have cumulative effects, especially on the immune and endocrine systems. Therefore, long-term toxicological studies and clinical trials are crucial to ensure the safety of selenium-enriched microbial products for widespread use. Currently, the regulation of selenium-enriched products in food and pharmaceuticals is primarily based on the Recommended Dietary Allowance (RDA) for selenium intake. The World Health Organization recommends a maximum daily selenium intake of 400 µg for adults [158], while the Chinese Dietary Guidelines suggest an intake range of 60 µg/day [159]. For clinical and food applications, it is essential to ensure that the selenium in microbial selenium-enriched products is primarily in safe organic forms, such as SeMet or SeCys, rather than the toxic Se(IV) form. Studies have shown that long-term intake of high doses of inorganic selenium or SeNPs (>4.3 µg/kg bw/day) may lead to adverse effects such as liver and kidney toxicity, and endocrine disruption [160]. Therefore, acute and chronic toxicological testing, in vivo biodistribution analysis, and tracking of metabolic products are critical for evaluating the safety of selenium-enriched microbial products. Future research could focus on using genetic engineering or fermentation process control to improve the particle size consistency and biocompatibility of SeNPs, thereby reducing toxicity risks.

Currently, the production cost of selenium-enriched microbial products remains high, mainly due to the cost of selenium sources and stringent microbial cultivation requirements. Optimizing culture media, enhancing selenium utilization efficiency, and minimizing energy consumption and raw material costs are essential for achieving industrial-scale production. Developing microbial strains with higher selenium absorption and conversion capacity could reduce dependence on expensive selenium sources, which would be a vital strategy for lowering production costs. Although microbial conversion can transform inorganic selenium into safer organic selenium forms, there are still differences in the absorption mechanisms and distribution of various selenium forms (such as SeMet, SeCys, and SeNPs) in the body. Organic selenium forms are more readily integrated into human proteins for utilization, whereas SeNPs, despite exhibiting excellent antioxidant properties, still lack clarity regarding their accumulation in the body, bio-conversion mechanisms, and long-term toxicity. Further evaluation is needed. In addition, the industrialization of microbial selenium-enriched fermentation faces challenges such as unstable yields, limited selenium accumulation levels, and high costs. Future development directions include the following: the development of high-selenium-tolerant engineered strains, utilizing selenium-rich waste materials as culture medium sources, and optimizing fermentation conditions to improve conversion rates.

Future research on selenium-enriched microbial products should focus on the application of multi-omics technologies, such as genomics, transcriptomics, and metabolomics, to better understand selenium-enrichment mechanisms and identify efficient selenium-conversion enzymes and key genes. Additionally, employing synthetic biology to develop novel strains with improved selenium absorption and conversion efficiency is crucial for enhancing product yields. Finally, integrating selenium-enriched microorganisms with other biotechnologies like nanotechnology and bioengineering could lead to more specialized, functional products, advancing both health and industrial applications. This multi-disciplinary approach will foster the development of advanced bioproducts with broader applications and environmental sustainability.

## Data Availability

No new data were created or analyzed in this study.
